# Elevated homocysteine levels in type 2 diabetes induce constitutive neutrophil extracellular traps

**DOI:** 10.1038/srep36362

**Published:** 2016-11-04

**Authors:** Manjunath B Joshi, Guruprasad Baipadithaya, Aswath Balakrishnan, Mangala Hegde, Manik Vohra, Rayees Ahamed, Shivashankara K Nagri, Lingadakai Ramachandra, Kapaettu Satyamoorthy

**Affiliations:** 1 School of Life Sciences, Manipal University, Manipal, India; 2 Department of Medicine, Kasturba Medical College, Manipal University, Manipal, India; 3 Department of Surgery, Kasturba Medical College, Manipal University, Manipal, India

## Abstract

Constitutively active neutrophil extracellular traps (NETs) and elevated plasma homocysteine are independent risk factors for Type 2 Diabetes (T2D) associated vascular diseases. Here, we show robust NETosis due to elevated plasma homocysteine levels in T2D subjects and increased components of NETs such as neutrophil elastase and cell free DNA. Cooperative NETs formation was observed in neutrophils exposed to homocysteine, IL-6 and high glucose suggesting acute temporal changes tightly regulate constitutive NETosis. Homocysteine induced NETs by NADPH oxidase dependent and independent mechanisms. Constitutively higher levels of calcium and mitochondrial superoxides under hyperglycemic conditions were further elevated in response to homocysteine leading to accelerated NETosis. Homocysteine showed robust interaction between neutrophils and platelets by inducing platelet aggregation and NETosis in an interdependent manner. Our data demonstrates that homocysteine can alter innate immune function by promoting NETs formation and disturbs homeostasis between platelets and neutrophils which may lead to T2D associated vascular diseases.

Constitutively active innate immune responses are associated with pathophysiological conditions of several diseases such as diabetes and obesity^1^, autoimmune^2^, neurodegenerative disorders^3^ and thrombosis related diseases^4^. As an essential component of innate immune system, neutrophils play an important role by inducing hyper-responsive pro-inflammatory milieu during pathogenesis of such diseases. Neutrophils also actively eliminate pathogens through various mechanisms such as phagocytosis, release of antimicrobial factors and through recently discovered phenomenon of producing extracellular traps (NETs)^5^. In response to myriad of pathogens and their products, cytokines and pharmacological inhibitors/inducers, neutrophils expel meshwork of histones and granular proteins bound chromatin outside the cells to produce extracellular traps through a process known as NETosis^5^,^6^,^7^,^8^. In addition to histones, protein component of NETs includes elastase, myeloperoxidase, cathepsin G, bactericidal permeability increasing protein and others bound to DNA backbone. Besides normal innate immune function of NETosis in eliminating pathogens, NETs have been shown to induce host tissue damage associated with autoinflammatory diseases^2^, preeclampsia^9^, Alzheimer’s diseases^10^, pancreatitis^11^ and cancer metastasis^12^. Previously, we have shown that constitutive production of NETs during hyperglycemic condition results in reduced responsiveness to lipopolysaccharide (LPS) in type 2 diabetes (T2D) subjects indicating as one of the causes of recurrent infections during diabetes^13^. Using diabetic and Padi4^−/−^ mouse models, Wong *et al*.^14^, have demonstrated hyperglycemia primed NETs were responsible for delayed wound healing^14^. Elevated levels of NETs components such as cell free DNA (cfDNA), nucleosomes and neutrophil elastase in plasma was positively correlated to diabetes associated nephropathy and cardiovascular diseases^15^. NETs have also been shown to possess pro-thrombotic and atherogenic properties in the context of vascular diseases such as atherosclerosis^16^,^17^ and stroke^18^.

Homocysteine is a thiol-containing amino acid intermediate of methionine synthesis which is used in the transmethylation reaction. Elevated levels of serum homocysteine has been demonstrated either as co-morbid condition or as one of the independent risk factor for developing cerebrovascular diseases including stroke, dementia and Alzheimer’s, coronary artery diseases, preeclampsia and diabetes^19^. Several studies have established an association of increased homocysteine levels in T2D with insulin resistance^20^ and with an increased risk for progression of vascular diseases^20^,^21^,^22^,^23^,^24^. Studies have shown association of genetic variations in key genes regulating homocysteine metabolism. Prominent causal genetic polymorphisms extensively studied are methylenetetrahydrofolate reductase (MTHFR; rs1801133), cystathionine-β-synthase (CBS; rs5742905), methionine synthase (MN; rs1805087), and nicotinamide N-methyltransferase (NNMT; rs694539)^19^. These mutations are attributed to aberrant homocysteine metabolism leading to its elevated levels in plasma. Poor dietary intake of folate, vitamin B6 and vitamin B12 are also associated with increased levels of serum homocysteine^25^.

Individuals with T2D are long known to be characterized by high incidence of vascular complications and elevated levels of serum homocysteine. We and others have reported constitutive activation of neutrophils leading to abundant NETs in plasma of T2D subjects^13^,^14^,^15^. We have also shown that excess glucose dampens the ability of neutrophils to form extracellular traps which mimics the conditions in individuals with uncontrolled diabetes^13^. NETs play a key role in the pathogenesis of vascular diseases and homocysteine is strongly associated with vascular complications. Therefore in the present study we show the effects and mechanisms of homocysteine on NETs formation and establish a functional relationship between constitutive NETosis, elevated homocysteine and platelet aggregation in diabetic individuals which may lead to increased risk for vascular diseases.

## Results

### High glucose stimulates NETosis and NETs associated components are significantly elevated in serum of T2D subjects

**Figure 1:**
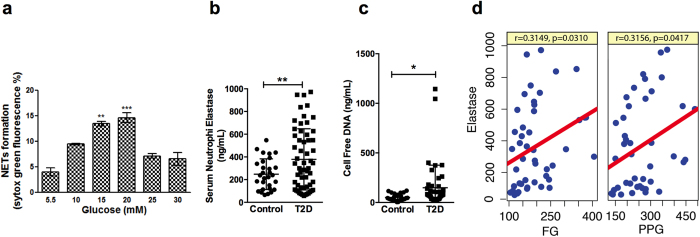
Influence of high glucose on NETosis. (**a**) Neutrophils isolated from peripheral blood (n = 5) was subjected to normal (5.5 mM) and high (indicated concentration) glucose for 24 hours and extracellular DNA was stained with SYTOX Green, followed by quantification in fluorimeter. Data is represented as percentage of maximal SYTOX Green fluorescence and significant accumulation of NETs due to high glucose is indicated as asterisk **p < 0.01, ***p < 0.001. Plasma from healthy control subjects (n = 31) and T2D subjects (n = 57) were screened for neutrophil elastase (**b**) and cell free DNA (**c**). Data are represented as scatter plots ± SEM and significant changes between control and diabetes subjects are represented as *p < 0.05, **p < 0.01. (**d**) Linear correlation plots between elastase and fasting glucose (FG) and post-prandial glucose (PPG) are shown. Pearson’s coefficient and p values are mentioned within the box.

As a first step, we examined the influence of glucose on neutrophils isolated from normal subjects. As reported earlier by us^13^ and others^14^,^15^, a concentration dependent increase in glucose levels (15 mM and 20 mM) significantly increased NETosis. Further increase in glucose concentration led to inhibition of NETosis (Fig. 1a). Our analysis in plasma of control (n = 31) and T2D (n = 57) subjects indicated significant elevation of neutrophil elastase and cfDNA in diabetic subjects. Mean concentration of plasma neutrophil elastase in healthy control was 248.2 ± 25.1 ng/ml and was significantly increased in diabetic individuals (378.6 ± 35.7 ng/ml; p 0.0085) (Fig. 1b). Similar to neutrophil elastase, plasma cfDNA levels were also elevated (148.7 ± 33.5 ng/ml; p 0.036) and showed nearly threefold increase in diabetic subjects compared to healthy controls (50.86 ± 7.0 ng/ml) (Fig. 1c). Correlation analysis indicated significant association between elastase and fasting glucose (r = 0.32; p = 0.031) (Fig. 1d) and with postprandial glucose (r = 0.32, p = 0.042) (Fig. 1d), suggesting potential involvement of hyperglycemia in diabetic subjects to induce constitutive NETosis.

### Homocysteine is a potential inducer of NETosis

**Figure 2:**
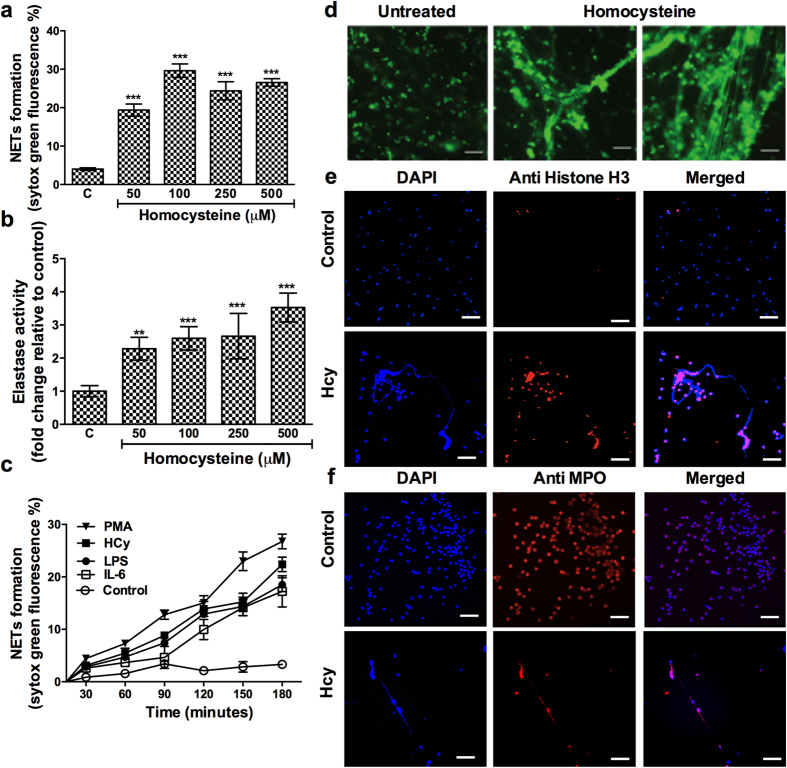
Homocysteine induces NETosis. Neutrophils isolated from peripheral blood from healthy donors were (n = 8) stimulated with indicated concentration of homocysteine for three hours. (**a**) NETs were quantified in fluorimeter upon staining extracellular DNA with SYTOX Green (5 μM) for 10 minutes in dark. Data is represented as percentage of maximal SYTOX Green fluorescence. (**b**) NETs induced by homocysteine were dismantled with DNase (8 U/ml) for 20 minutes and NETs associated elastase was measured after reacting with fluorescence tagged elastase substrate. Fold change in elastase activity with respect to untreated control is shown. Statistically significant induction of NETs (**a**) and elastase activity (**b**) compared to untreated control is represented by asterisk **p < 0.01, ***p < 0.001. (**c**) Freshly isolated neutrophils were treated with Homocysteine (Hcy) (100 μM), IL-6 (25 ng/ml), PMA (20 ng/ml), LPS (2 μg/ml) and kinetics of NETs formation was recorded upon staining with SYTOX Green at different time intervals in plate reader. (**d**) Representative micrographs of neutrophils treated in presence or absence of homocysteine (100 μM) for three hours and stained with SYTOX Green. Scale bar 100 μm. Control and homocysteine induced neutrophils were examined for presence of citrullinated histone (red) and DNA (blue) (**e**); myeloperoxidase (red) and DNA (blue) (**f**). Scale bar 200 μm.

To test the hypothesis that homocysteine might induce NETs formation, we treated freshly isolated peripheral neutrophils with different concentrations of homocysteine and examined for NETs formation by qualitative and quantitative analysis of DNA lattices and neutrophil associated elastase. We found homocysteine induced NETosis profoundly in a concentration dependent manner reaching plateau at 100 μM and above (Fig. 2a). Enzyme assays indicated concentration dependent increase in elastase activity when neutrophils were stimulated with homocysteine. At a minimum concentration of 50 μM of homocysteine, elastase activity was enhanced by three fold and was further increased up to five fold at a maximum concentration of 500 μM (Fig. 2b). We then compared the ability of homocysteine to activate NETs with other inducers of NETosis. A time course analysis revealed early response of neutrophils to phorbol myristate acetate (PMA) (45–60 minutes) and produced abundant NETs at the end of three hours compared to other inducers such as homocysteine, IL-6 and lipopolysaccharide (LPS). Although homocysteine, IL-6 and LPS begin to produce NETs by 60 minutes, homocysteine was more potent in releasing DNA lattices than IL-6 or LPS (Fig. 2c). These results suggests that NETs release was inducer dependent and homocysteine was as robust as PMA when compared to weak inducers such as ionomycin and IL-8^26^,^27^. We further characterized homocysteine induced NETs by staining for histone post-translational modifications (citrullinated histone H3) and other granular proteins such as myeloperoxidase by immunocytochemistry. We observed co-localization of DNA lattices along with both citrullinated histones (Fig. 2e) and myeloperoxidase (Fig. 2f).

### Constitutive activation of NETs formation during diabetic conditions positively correlates increased plasma homocysteine

**Figure 3:**
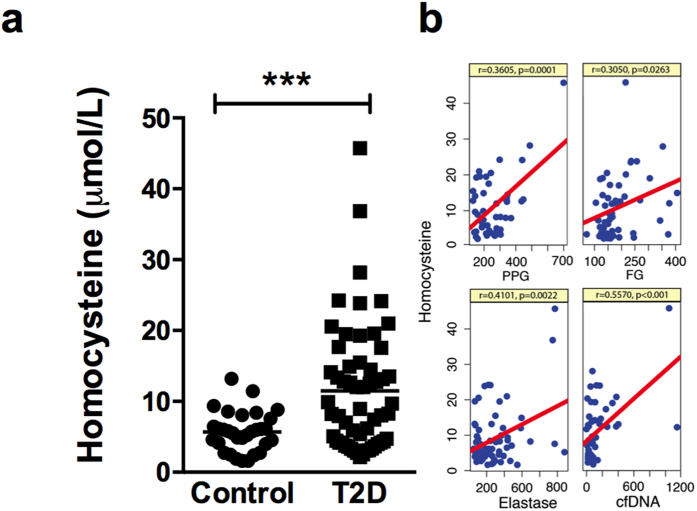
Plasma homocysteine levels are elevated in T2D and positively correlate with components of NETs. (**a**) Plasma homocysteine levels were measured in control (n = 31) and T2D (n = 57) subjects. Data are represented as scatter plots ± SEM and significant changes between control and T2D subjects are represented as ***p < 0.001. (**b**) Linear correlation plots between homocysteine and either with postprandial glucose; fasting glucose; elastase or cfDNA are shown. Pearson’s coefficient and p values are mentioned within the box.

We next determined the plasma homocysteine levels in control and diabetic cohorts. HPLC based analysis revealed nearly two fold elevation of plasma homocysteine in diabetes subjects as opposed to healthy controls (Fig. 3a). The absolute mean of fasting plasma homocysteine concentration in healthy subjects and diabetic individuals were 5.76 ± 0.50 μmol/L (95% CI, 4.68–6.73) and 11.47 ± 18 μmol/L (95% CI, 9.113–13.84) respectively. Pearson’s correlation analysis revealed significant positive correlation between fasting glucose and homocysteine levels in individuals with diabetes (r = 0.305, p = 0.026) (Fig. 3b). Similarly, correlation between postprandial glucose and homocysteine levels was found to be significant (r = 0.36, p = 0.0001) (Fig. 3b), indicating hyperglycemia influences homocysteine levels. Correlation analysis between plasma homocysteine and neutrophil elastase levels revealed significant positive association (r = 0.41, p = 0.0023) (Fig. 3b). Similar to plasma elastase, cfDNA levels also showed positive association with homocysteine (r = 0.56, p = 1.476e-05) (Fig. 3b). Such an increase in homocysteine levels in diabetic subjects and its strong correlation with NETs components suggested a plausible causal relationship with constitutive NETosis during diabetes.

### Homocysteine accelerates hyperglycemia induced NETs during T2D

**Figure 4:**
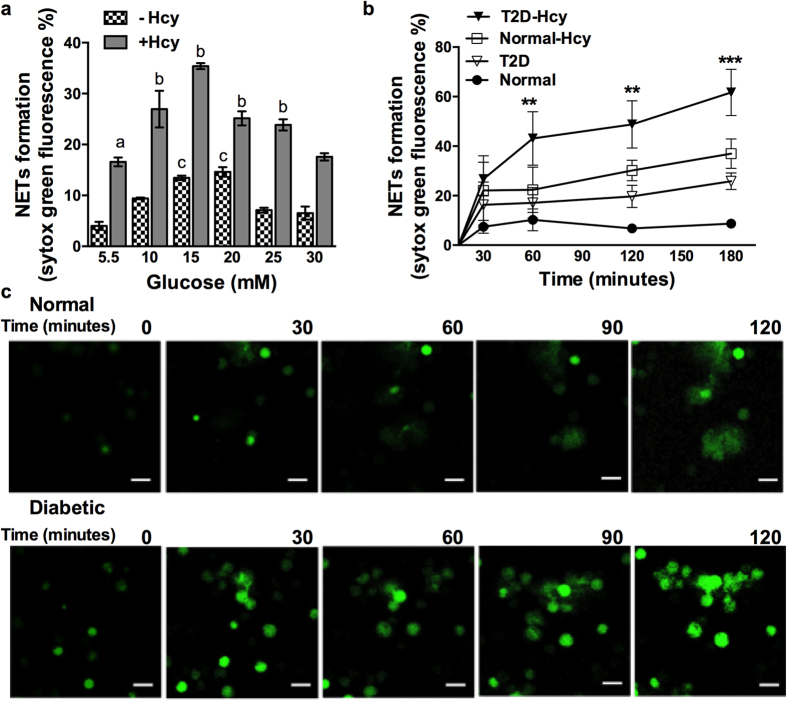
Homocysteine induced NETosis exhibits synergistic effects with hyperglycemia. (**a**) Neutrophils were cultured under normal glycemic (5.5 mM glucose) or hyper glycemic (indicated concentration of glucose) for 24 hours and followed by activation with homocysteine (100 μM) for further 3 hours. NETs were measured after staining DNA with SYTOX Green in fluorimeter. Statistical significance in modulation of NETosis under various conditions are denoted as follows, (**a**) = significant accumulation of NETs (atleast p < 0.001) in homocysteine treated cells compared to untreated control; (**b**) = significant increase in NETosis (atleast p < 0.01) in homocysteine treated cells under hyperglycemic conditions compared to that of treated under normoglycemia; (**c**) = significant production of NETs (atleast p < 0.01) due to high glucose compared to untreated control. Data of three independent experiments from healthy volunteers (n = 4) is shown. (**b**) Neutrophils from healthy subjects (n = 10) and diabetic subjects (n = 10) were isolated and treated with homocysteine (100 μM) and accumulation of NETs were measured at different time points as indicated after staining with SYTOX Green. Data is represented as percentage of maximal SYTOX Green fluorescence ± SEM. Statistical significance in increased NETosis between homocysteine treated control and diabetic neutrophils is represented as **p < 0.01, ***p < 0.001. (**c**) Peripheral neutrophils isolated from normal and diabetic individuals were treated with homocysteine (100 μM) along with SYTOX Green. Images captured at indicated time points are given. Scale bar 10 μm.

We have earlier reported constitutive pre-activation of neutrophils in diabetes individuals leading to decreased response to LPS^13^. Therefore, we tested influence of simulated hyperglycemic condition on homocysteine induced NETs in neutrophils incubated with different concentrations of glucose (Fig. 4a). When treated with glucose at a concentration range of 10–25 mM together with homocysteine, neutrophils exhibited synergistic effect on NETs formation. To investigate further and to understand physiological relevance of combined effect of homocysteine and glucose in diabetic individuals, we performed a time course analysis of homocysteine induced effect on NETosis. We found that NETs were constitutively produced in neutrophils isolated from diabetic individuals as opposed to normal healthy controls (Fig. 4b). In response to homocysteine, neutrophils from diabetic subjects expelled NETs more rapidly and robustly (within 45–60 minutes) and were more abundant than the controls at the end of 180 minutes (Fig. 4b). These results suggest that hyperglycemia along with elevated homocysteine levels in diabetic individuals were responsible for constitutive NETosis. Further, accelerated NETosis due to homocysteine in diabetic conditions was also observed by time-lapse video microscopy (Fig. 4c).

### Homocysteine induced NETosis requires reactive oxygen species (ROS) derived from both NADPH oxidase and mitochondria

**Figure 5:**
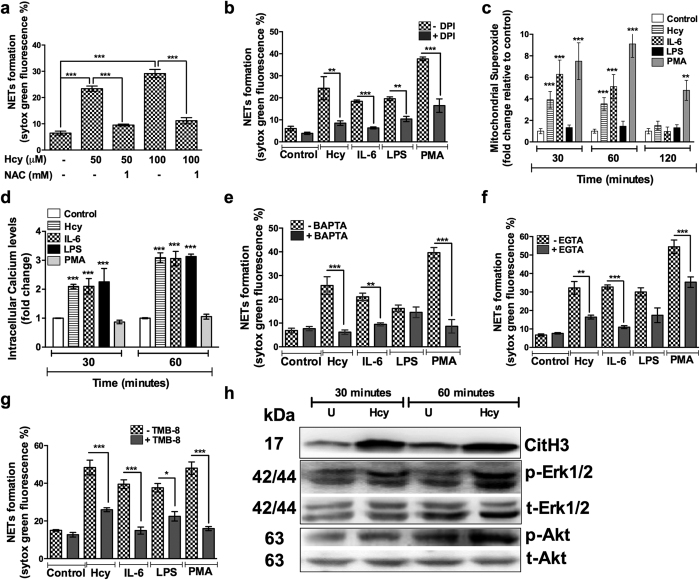
Homocysteine induced NETosis is ROS and Ca2 + influx dependent. Freshly isolated peripheral neutrophils were pretreated with either N-acetyl cysteine (1 mM) (**a**) or Diphenyleneiodonium (20 μM) (**b**) or BAPTA (10 μM) (**e**) or EGTA (10 mM) (**f**) or TMB-8 (20 μM) (**g**) for 30 minutes and followed by activation either in presence or absence of homocysteine alone (**a**) or homocysteine (100 μM) IL-6 (25 ng/ml), PMA (20 ng/ml), LPS (2 μg/ml) for three hours. Extent of NETs formation was measured after staining with SYTOX Green (5 μM) in fluorimeter. Data is represented as percentage of maximal SYTOX Green fluorescence. Statistically significant modulation in NETosis is denoted by asterisk ***p < 0.001, **p < 0.01. Neutrophils were loaded with MitoSOX Red (5 μM) (**c**) or Fluo3-AM (5 μM) (**d**) for 30 minutes in dark, followed by activation with different inducers for indicated time points. After a gentle cell wash with PBS, mitochondrial superoxides and intracellular Ca^2+^ were measured using flow cytometry and fluorescence plate reader respectively. Data is represented as fold change relative to untreated control for each time point. (**h**) Neutrophils were treated with homocysteine for indicated time points and lysed. Cell lysates were processed for immunoblotting and probed for citrullinated histone, phospho Erk^Thr202/Tyr204^ and total Erk; phospho Akt^ser473^ and total Akt.

We next performed following series of experiments to understand the mechanisms involved in homocysteine induced NETs formation. Since NETosis has been demonstrated to be reactive oxygen species (ROS) dependent^28^, inclusion of pan-antioxidant N-acetyl cysteine (NAC) abrogated homocysteine induced NETs formation (Fig. 5a). To examine the specificity of NADPH oxidase on homocysteine induced NETs, NETosis assays were performed in presence of diphenyleneiodonium (DPI), a NADPH oxidase inhibitor. We observed that DPI (20 μM) inhibited homocysteine induced NETs formation by 50%. DPI also reduced the effects of IL-6, LPS and PMA induced NETs. However, the DPI effects were more potent on PMA and IL-6 (p < 0.001) induced NETs when compared to homocysteine (p < 0.01) suggesting alternate pathways and mechanisms may exist to drive homocysteine stimulated NETosis (Fig. 5b). Besides, DPI is also known to inhibit mitochondrial superoxide radicals^29^. Thus, we tested mitochondrial superoxide levels in homocysteine treated neutrophils along with other inducers. Mitochondrial superoxides were detectable by 30 minutes and PMA induced rapid and more sustained free radicals when compared to homocysteine and IL-6 (Fig. 5c). While the superoxide levels continued to increase in PMA treated cells, the homocysteine and IL-6 induced superoxides levels begin to decline at 60 minutes and returned to basal levels by 120 minutes. These results suggest that both the kinetics and quantum of mitochondrial superoxide production vary among the activators of NETosis.

### Homocysteine induced NETosis involves calcium (Ca^2+^) influx

Previously, several studies have demonstrated the involvement of Ca^2+^ influx in NADPH oxidase independent mechanism of NETosis^30^,^31^. Therefore, we first examined the influence of NETs activators on Ca^2+^ influx. Quantitative measurement of intracellular Ca^2+^ using Fluo3-AM indicated homocysteine, IL-6 and LPS robustly induced Ca^2+^ influx (Fig. 5d). We also observed a two fold increase in Ca^2+^ accumulation at 30 minutes which was further elevated to three fold at 60 minutes. PMA did not induce Ca^2+^ mobilization in these conditions. To evaluate influence of Ca^2+^ mobilization on NETs formation, neutrophils were stimulated with various activators in presence of calcium chelators EGTA (extracellular) and BAPTA (intracellular) and antagonist of intracellular calcium mobilization (TMB-8). Both intracellular (Fig. 5e) and extracellular (Fig. 5f) chelation of Ca2^+^ resulted in significant inhibition of NETosis activated by homocysteine, IL-6 and PMA. However, NETs formed by LPS were not affected. Inhibition of intracellular calcium release with TMB-8 significantly blunted the NETosis induced by homocysteine, IL-6 and PMA (Fig. 5g). LPS activated NETs were minimally inhibited by TMB-8. These results clearly suggested distinct role of Ca^2+^ for NETosis induced by homocysteine and LPS.

### Homocysteine activates Akt and Erk1/2 phosphorylation and histone citrullination in neutrophils

Earlier studies have demonstrated activation and nuclear translocation of peptidyl arginine-deaminase 4 (PAD4) and subsequent citrullination of histones leading to chromatin decondensation^30^,^31^. A time course analysis indicated homocysteine facilitated citrullination of histone within 30 minutes and further increased at 60 minutes (Fig. 5h). Activation and requirement of Akt and Erk1/2 have been demonstrated during NETs formation^32^,^33^. To understand underlying signaling mechanisms for homocysteine induced NETs, we examined activation of Akt and Erk kinases. Immunoblotting analysis showed nearly two fold increase in both Erk1/2 ^Thr202/Tyr204^ and Akt ^ser473^ phosphorylation within 60 minutes post stimulation with homocysteine (Fig. 5h).

**Figure 6:**
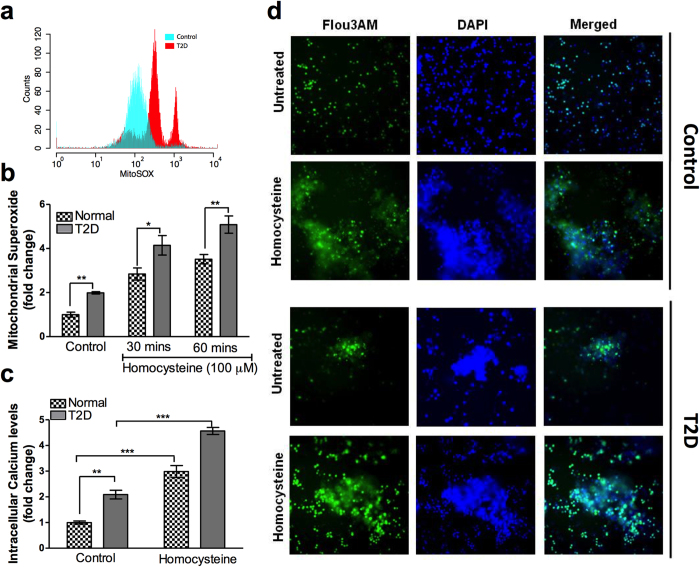
Neutrophil from T2D subjects contains increased constitutive levels of mitochondrial superoxide and intracellular Ca^2+^. (**a**) Representative flow cytometry analysis showing constitutive mitochondrial superoxide levels in normal and T2D subjects loaded with mitoSOX Red (5 μM) for 30 minutes. Neutrophils isolated from normal (n = 4) and diabetes subjects (n = 4) were either loaded with MitoSOX Red (5 μM) (**b**) or Fluo3-AM (5 μM) (**c**) for 30 minutes in dark, followed by stimulation with homocysteine (100 mM) indicated time points. Mitochondrial superoxides and intracellular Ca^2+^ were measured using flow cytometry and fluorescence plate reader respectively. Data is represented as fold change relative to untreated control of normal individuals. Significant changes are denoted by asterisk *p < 0.05, **p < 0.01, p < 0.001). Representative fluorescence microscopy images (**d**) showing constitutive and homocysteine (100 μM for 3 hours) induced Ca^2+^ levels in neutrophils isolated from control and T2D subject. Ca^2+^ is stained using Fluo3-AM (Green) and co-stained with nuclear dye DAPI (1 μg/ml) for 30 minutes.

Our data showed homocysteine induced NETs were mitochondrial superoxide and Ca^2+^ influx dependent. Therefore we examined constitutive and inducible levels of mitochondrial superoxide and Ca^2+^ in T2D subjects. Flow cytometry based analysis revealed significantly higher constitutive levels of mitochondrial superoxide in neutrophils from T2D subjects (Fig. 6a). Constitutive levels of mitochondrial superoxide in neutrophils isolated from T2D subjects were elevated and homocysteine accelerated superoxide production in a time dependent manner in diabetic individuals by 6 fold compared to 4 fold in normal individuals (Fig. 6b). Similarly, we observed constitutively increased Ca^2+^ levels in neutrophils isolated from T2D subjects. The constitutive NETs formation in neutrophil prepared from diabetic individuals were associated with Ca^2+^ accumulation, suggesting involvement of elevated Ca^2+^ in inducing NETs (Fig. 6d). Further, treatment with homocysteine increased Ca^2+^ influx in neutrophils from T2D subjects when compared to normal subjects (Fig. 6c). Taken together, our data suggests increased constitutive levels of Ca^2+^ and mitochondrial superoxide might serve as feed forward mechanism for the NETosis induced by increased homocysteine in diabetic condition.

### Increased serum IL-6 in diabetes positively correlates with homocysteine and neutrophil elastase

**Figure 7:**
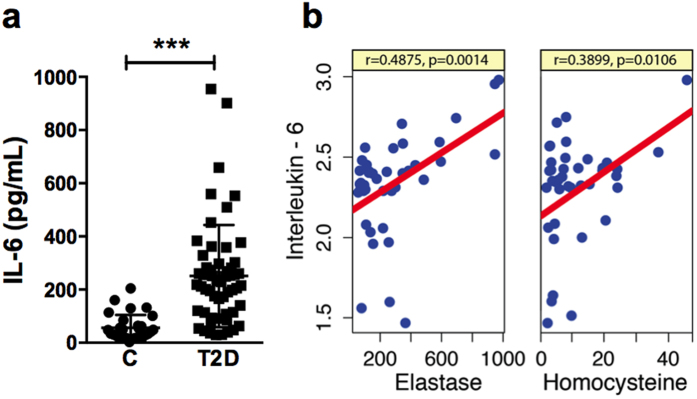
Elevated IL-6 in diabetes positively correlates with neutrophil elastase and homocysteine. (**a**) Scatter plot showing plasma IL-6 levels in control and diabetes subjects. Statistically significant increase in diabetes cohort is represented by ***p < 0.001. (**b**) Linear correlation analysis between IL-6 and either with elastase or homocysteine are given. IL-6 values are log 10 transformed. Pearson’s coefficient and statistical significance are indicated by r and p respectively.

Increased systemic IL-6 in diabetes is well known and we have shown that IL-6 is a potent inducer of NETs^13^. We observed that plasma IL-6 levels were nearly fivefold elevated in diabetes subjects (251.0 ± 25.65 pg/ml) when compared to healthy subjects (56.40 ± 8.87 pg/ml) (Fig. 7a). We also found a strong correlation between IL-6 and homocysteine levels in diabetes. A correlation analysis showed direct association between plasma homocysteine and IL-6 (r = 0.49, p = 0.00079) (Fig. 7b). Interestingly, IL-6 concentrations also directly correlated with neutrophil elastase levels in (r = 0.707, p = 3.282e-07) (Fig. 7b) diabetic group. Our data indicated possible interplay between homocysteine and IL-6 during diabetes thus influencing NETosis. We performed stepwise multiple regression analysis to understand influence of homocysteine, IL-6 and fasting glucose together or in different combinations on NETs components in plasma (Table No 1). Influence of homocysteine and IL-6 was more effective in diabetic environment characterized by increased fasting glucose levels. Co-efficient of determination (R^2^) values were significantly diluted when whole study cohort was considered, suggesting homocysteine and IL-6 induced NETs were enhanced in hyperglycemic conditions. Interestingly, in control cohort, IL-6 showed significant correlation with neutrophil elastase and cfDNA but there was no influence of homocysteine on NETs components.

### IL-6 release by neutrophils is elevated by homocysteine

**Figure 8:**
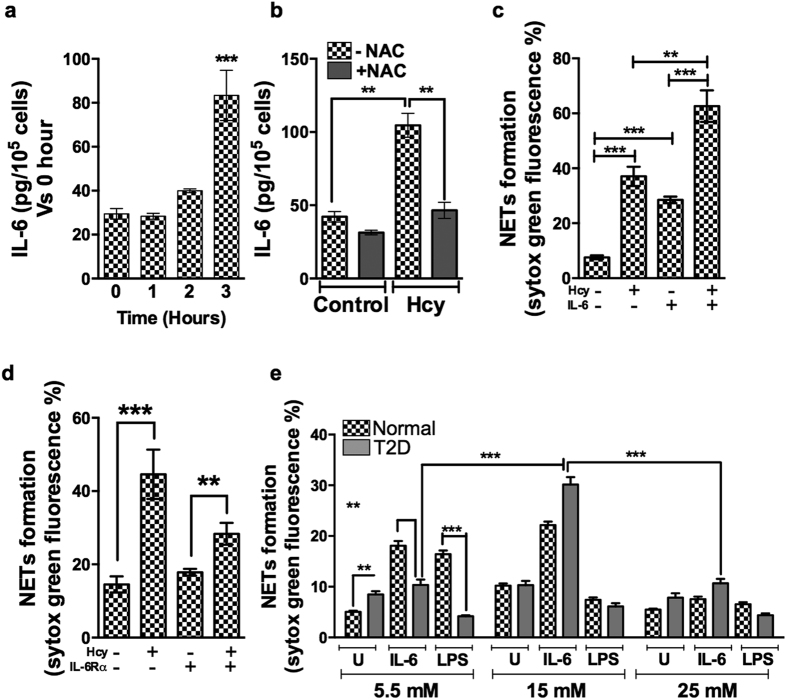
Influence of homocysteine on IL-6 secretion from neutrophils and on NETosis in diabetes. Freshly isolated neutrophils were treated with homocysteine (100 μM) for indicated time points (**a**) or in presence of N-acteyl cysteine (1 mM) (**b**) for 3 hours. IL-6 protein levels were measured in conditioned medium upon centrifugation by ELISA. Significant levels of IL-6 secreted (pg/10^5 ^cells) compared to controls are represented by **p < 0.01, ***p < 0.001. Neutrophils were either treated with homocysteine (100 μM) or IL-6 (25 ng/ml) or both together (**c**) or in presence or absence of IL-6R neutralizing antibody (2 μg/ml) (**d**) for three hours and NETs were measured by SYTOX Green fluorescence. (**e**) Peripheral neutrophils from normal (N = 4) and diabetic (N = 4) were cultured in varied concentrations of glucose as indicated and treated either with LPS (2 μg/ml) or IL-6 (25 ng/ml) for 3 hours. Significant differences between different groups are indicated by asterisk **p < 0.01, ***p < 0.001.

Neutrophils are rich source of IL-6 and both elevated homocysteine and IL-6 levels have been demonstrated in diabetes associated pathologies. We show that treatment of neutrophils by homocysteine led to elevated IL-6 secretion by 3 hours and its concomitant effects on NETosis (Fig. 8a) and was inhibited by antioxidant N-acteyl cysteine, suggesting that homocysteine mediated IL-6 release is ROS dependent (Fig. 8b). To test influence of IL-6 and homocysteine together on NETosis, neutrophils were stimulated either alone or in combination with homocysteine and IL-6. NETs produced by the combined IL-6 and homocysteine treatment were additive and significantly higher than with IL-6 or homocysteine alone (Fig. 8c). As IL-6 is a known inducer of NETosis, we tested whether homocysteine induced IL-6 release has any autocrine effects on NETosis. Inclusion of neutralizing antibody against IL-6R decreased the levels of NETs induced by homocysteine (Fig. 8d), suggesting that homocysteine facilitated IL-6 secretion regulate NETosis by an autocrine effect. Since homocysteine accelerated NETosis in diabetic subjects (Fig. 4b) and our earlier study has revealed impeded NETosis in T2D subjects in response to LPS, we investigated consequence of IL-6 induced NETosis in neutrophils from diabetic individuals cultured under various concentrations of glucose. Besides reproducing our earlier data of LPS response on NETosis from diabetes individuals, we also observed varied effect of IL-6 under different glucose concentrations (Fig. 8e). Diabetic neutrophils did not make additional NETs when cultured in either normal (5.5 mM) or higher (25 mM) glucose conditions in response to IL-6; however, under moderately elevated glucose (15 mM) culture conditions, significant increase in NETosis was observed. Therefore rate of IL-6 induced NETosis may depend on the glucose levels during diabetic conditions.

### Homocysteine activated platelets induce NETosis

**Figure 9:**
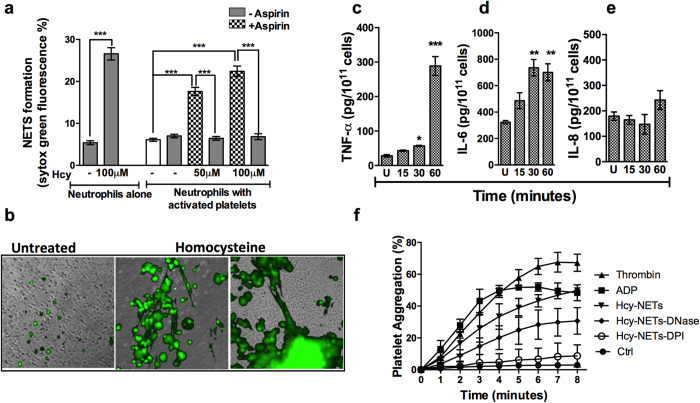
Homocysteine induces bidirectional activation of neutrophils and platelets. (**a**) Platelets were isolated from peripheral blood from healthy subjects (n = 4) and treated with or without homocysteine alone or in presence of aspirin (20 μM) for 30 minutes and washed with PBS. Activated platelets (10^4^) were co-cultured with neutrophils (10^5^) from same individual for 3 hours. Neutrophil NETs were stained with SYTOX Green (5 μM) and quantified in fluorimeter. Data of three independent experiments is represented as maximal SYTOX Green fluorescence ± SEM. (**b**) Representative images of co-culture of homocysteine activated platelets and neutrophils stained with SYTOX Green. Freshly isolated platelets (n = 3) were stimulated with or without homocysteine for indicated time points and supernatant were analyzed for TNF-α (**c**); IL-6 (**d**) and IL-8 (**e**) by ELISA. Significant accumulation of cytokines with respect to untreated control (U) are indicated by asterisk *p < 0.05, **p < 0.01, ***p < 0.001. (**f**) Neutrophils isolated from peripheral blood (n = 4) were stimulated with homocysteine for 30 minutes, washed gently by centrifugation and cultured with fresh medium for 2 hours in presence or absence of DNase (8 U/ml) or DPI (20 μM). Conditioned medium was collected by brief centrifugation (800 rpm for 5 minutes) and treated with platelets in 96 well plate and absorbance was measured at for indicated time points. ADP (10 μM) and Thrombin (5 U/ml) served as positive control for platelet aggregation. Data is represented as percentage of platelet aggregation.

Increased plasma homocysteine has been demonstrated as one of the risk factors to activate platelets and these subsequently aggregate in the context of thrombosis associated diseases^34^. In the sepsis model, Clark *et al*.^35^ showed platelets upon stimulation with LPS activated TLR4 expression leading to platelet-neutrophil interaction which subsequently resulted in NETosis^35^. We next examined the effects of platelets that are activated with homocysteine on NETs formation. Co-culture of neutrophils with platelets stimulated with homocysteine led to significant accumulation of NETs within three hours (Fig. 9a). Induction of NETs through homocysteine activated platelets was concentration dependent (100 μM > 50 μM) and was significantly abrogated upon inclusion of aspirin, a potent platelet aggregation inhibitor. Microscopy analysis also showed that homocysteine activated platelets facilitated NETosis (Fig. 9b). Next, we sought to determine presence of cytokines such as TNF-α, IL-6 or IL-8 in homocysteine activated platelet secretome which are known potent inducers of NETs. ELISA based assays revealed homocysteine induced significant release of TNF-α (288 ± 27.3 pg/ml) compared to untreated control (28.12 ± 3.36 pg/ml) (Fig. 9c). Nearly two fold increase in IL-6 (700.56 ± 64.33 pg/ml) in homocysteine treated platelets was found against untreated control (323.16 ± 12.37 pg/ml) (Fig. 9d). However, homocysteine treatment did not facilitate significant IL-8 secretion (Fig. 9e). As NETs have been shown to induce platelet aggregation in thrombosis associated disorders, we tested for homocysteine induced NETs that might lead to platelet aggregation using ADP and thrombin as controls. We observed supernatant from neutrophil conditioned medium containing NETs induced platelet aggregation which was comparable with that of ADP and thrombin (Fig. 9f). Inclusion of DNase in presence of homocysteine significantly impeded platelet aggregation suggesting requirement of intact NETs. However DPI treated neutrophils failed to aggregate the platelets suggesting that neutrophil secretome *per se* do not induce platelet aggregation and that NETs were pre-requisite for such a phenomenon.

## Discussion

Our study provides the first evidence for homocysteine as a potential inducer of NETosis and its possible implications in diabetes and associated vascular complications. We show that (a) neutrophils from healthy volunteers responded to homocysteine to produce extracellular traps in a concentration and time dependent manner, (b) T2D individuals showed increased levels of serum homocysteine which were strongly correlated with neutrophil elastase and cfDNA, (c) homocysteine activates both NADPH oxidase dependent and independent (Ca^2+^ influx and mitochondrial superoxide) mechanisms leading to NETosis, (d) in presence of homocysteine, glucose primed neutrophils respond additively to produce NETs and (e) pro-inflammatory status and elevated levels of molecules such as IL-6 during diabetes act cooperatively with homocysteine to produce NETs. Homocysteine dependent NETs formation also distinguishes itself from other NETs inducing agents such as LPS by its requirement for Ca^2+^ and mitochondrial superoxide. Finally, we show that homocysteine can induce NETosis by (a) direct activation of neutrophils and (b) indirectly through the activation of platelets.

Homocysteine above the physiological levels exerts diverse deleterious effects on vascular and immune cells through synthesis of ROS leading to endothelial injury, platelet activation, smooth muscle cell proliferation, oxidation of LDL and induction of endothelial-monocyte interactions^36^,^37^. Influence of homocysteine on neutrophils is less understood. In rat neutrophils, Bryushkova *et al*.^38^, showed expression of NMDA receptors and subsequent oxidative burst in response to homocysteine^38^. Homocysteine induced superoxides *via* NADPH oxidase leading to activation of Erk1/2 resulting in chemotaxis and migration of human peripheral neutrophils^39^. Requirement of Akt and Erk1/2 signaling in inducing NETs have been demonstrated earlier^32^,^33^. Our analysis showed an induction of Erk1/2 and Akt phosphorylation in response to homocysteine in neutrophils.

NETosis is reactive oxygen species (ROS) dependent^40^ and based on the requirement of NADPH oxidase, it has been classified as NADPH oxidase dependent or independent phenomenon. Chemical inducers such as PMA, LPS and microbes (*S. aureus* and *Aspergillus sp*.) that induce NETs are NADPH oxidase dependent and are abrogated upon pharmacological inhibition of the enzyme^28^,^41^,^42^. Mutations in gene(s) coding for NADPH oxidase subunit proteins leads to chronic granulomatous disease and these patients fail to make NETs^43^. Contrary to this, Ca^2+^ ionophore induced NETs are demonstrated to be NADPH oxidase independent^27^,^44^. However in a recent study, ionomycin and IL-8 induced NETs were abolished in presence of NADPH oxidase antagonist DPI^26^. We found homocysteine activated NETs were blocked by DPI, although extent of inhibition in homocysteine treated neutrophils was lesser (p < 0.01) than that of PMA and IL-6 (p < 0.001). NADPH oxidase independent NETosis involves activation of mitochondrial superoxides. We observed mitochondrial superoxides accumulated within thirty minutes in neutrophils treated with homocysteine, IL-6 and PMA unlike LPS. However, Douda *et al*.^27^, observed that only ionomycin but not PMA treated cells were able to activate mitochondrial superoxides^27^. Taken together, we observed a requirement of both NADPH oxidase dependent and independent (mitochondrial ROS) mechanisms for homocysteine activated NETs. Recent studies have shown that NETs induced by *Cryptosporidium parvum*^45^ and a lipid mediator hepoxilin A3^46^ followed similar trend of activation by both NADPH oxidase dependent and independent pathways.

Role of Ca^2+^ mobilization in PAD4 mediated histone citrullination is required for the induction of NETs^26^,^27^. Homocysteine induced NETs were associated with robust citrullinated histones suggesting requirement of calcium in homocysteine induced NETosis. Inclusion of BAPTA and EGTA, potential Ca^2+^ influx inhibitors and TMB-8, inhibitor of intracellular calcium release abrogated NETs formed by homocysteine, IL-6 and PMA, but not in LPS treated neutrophils. Involvement of Ca^2+^ influx in PMA induced NETs is still remains elusive. Inclusion of EGTA (10 mM)^26^ and BAPTA (10 and 20 μM)^44^ abolished NETs induced by PMA. On the other hand, neutrophils cultured in presence of Ca^2+^ activated potassium channel of small conductance (SK channel) inhibitor NS8593 did not abrogate effects of PMA on NETs, instead increased PMA induced NETosis^27^.

We found significant elevation of plasma homocysteine in diabetic subjects compared to healthy controls. Various studies have established association between elevated levels of homocysteine and diabetes. Recent meta-analysis based on Mendelian randomization approach in 4011 diabetic cases and 4303 controls, revealed a strong evidence of causal association between homocysteine levels and T2DM^47^. We observed homocysteine levels in diabetic cohort positively correlated with neutrophil elastase and cfDNA, suggesting influence of homocysteine or IL-6 on constitutive NETosis in these individuals. Increase in NETs associated products such as neutrophil elastase and cfDNA in diabetes subjects were in agreement with a report from an earlier study^15^. Homocysteine showed synergistic effect on glucose (hyperglycemia) induced NETosis both in *in vitro* hyperglycemia model and in neutrophils isolated from T2D individuals. High glucose levels induced NETosis and NETs formation was further enhanced upon inclusion of homocysteine. This was unlike the effect of LPS under hyperglycemic conditions^13^ wherein the response to LPS was inhibited in neutrophils cultured in presence of high glucose. Therefore, selective activation of NETs in diabetic condition is based on inducer and in the context of either infection or sterile inflammation. Hence, we hypothesize homocysteine and LPS may follow different pathways for NETs formation. We found elevated levels of constitutive Ca^2+^ and mitochondrial superoxide in neutrophils isolated from diabetic individuals compared to control subjects as reported in earlier studies^48^,^49^. Interestingly, homocysteine further enhanced the Ca^2+^ influx and superoxide accumulation which might be responsible for accelerated NETosis in T2D neutrophils in response to homocysteine. Recent study conducted by Fadini *et al*.^50^, demonstrated spontaneous NETs in diabetic wounds and showed impaired inducible NETosis in response to both PMA and ionomycin^50^. Riyapa *et al*.^51^, demonstrated impaired NETosis in diabetes subjects induced by *Burkholderia pseudomallei*^51^. On the other hand, Wong and colleagues (2015) demonstrated hyperglycemia primed neutrophils produced robust NETs in response to ionomycin and PMA^14^. Neutrophils cultured at moderately higher levels of glucose (10–20 mM) and subsequent stimulation by homocysteine and IL-6 formed robust NETs and was inhibited at higher levels of glucose (25–30 mM). However, LPS failed to produce NETs in presence of glucose. NETs components (elastase and cfDNA) did not correlate with HbA1c (Fig. S1) suggesting acute higher levels of glucose might directly influence constitutive NETosis. Hence, distinct mechanisms for inducer dependent effects on NETosis and transient dynamics of glucose levels in diabetic subjects might also strongly influence the priming and also (in)activation of neutrophils and is context dependent.

Diabetes is associated with constitutive low grade inflammation characterized by elevation of pro-inflammatory cytokines such as IL-6 and IL-6 is a potent inducer of NETs^13^. We observed homocysteine increased secretion of IL-6 in neutrophils which was blocked by antioxidant N-acteyl cysteine. In the context of neurodegenerative diseases, in THP-1 monocytes, homocysteine elevated ROS dependent expression of IL-6, IL-1β, TNF-α and transglutaminase^52^. We observed an additive effect of IL-6 and homocysteine on NETosis. Analysis of plasma of diabetic subjects revealed elevated IL-6 and a positive correlation between IL-6 and homocysteine (r = 0.49, p = 0.00079) along with IL-6 and neutrophil elastase (r = 0.707, p = 3.282e-07). Several studies have demonstrated strong correlation between serum IL-6 and homocysteine in various diseases including diabetes^53^ and preeclampsia^54^. Interestingly T2D^13^,^14^,^15^ and preeclampsia^9^ are associated with increased NETosis. Elevated homocysteine together with increased IL-6 might further aggravate pro-inflammatory milieu in diseases associated with chronic/sterile inflammation.

Homocysteine in its various forms (oxidized, reduced) or thiolactone; and hydrogen sulfide influences blood coagulation, platelet activation and fibrinolysis. Homocysteine exerts pro-coagulant activity on platelets by stimulating Factor V and increasing expression of tissue factor in addition to inactivate anticoagulants such as thrombomodulin and protein C^34^,^55^. In a co-culture system, we observed that platelets activated with homocysteine induced NETs. Inclusion of antiplatelet drug aspirin abrogated the effect of activated platelets on NETosis, suggesting strong influence of homocysteine on biphasic activation of platelets and neutrophils leading to increased NETosis during diabetes. It has been noted that more than 300 active substances are released upon platelet activation^56^ including several cytokines such as TNF-α, IL-6 and IL-8 which are potent inducers of NETosis^9^,^13^,^57^. Analysis of platelet conditioned media revealed significant accumulation of TNF-α and IL-6 in response to homocysteine, suggesting elevated homocysteine might activate platelets to release proinflammatory cytokines responsible for constitutive NETs production and exacerbate the inflammatory milieu. Interestingly, we found that NETs significantly facilitated platelet aggregation which was inhibited by inclusion of DNase and DPI, suggesting a bidirectional activation of neutrophils and platelets during thrombosis related diseases. NETs have been demonstrated in the pathogenesis of both arterial and venous thrombosis. Fuchs *et al*.^58^ demonstrated NETs in the blood upon perfusion resulted in platelet activation, aggregation and recruitment of RBC and fibrin for clotting. NETs were enriched in thrombus in Baboon DVT model which was overcome by the addition of DNase, suggesting NETs can potentially cause thrombosis^58^. In a cross sectional cohort of 282 suspected coronary artery disease subjects, dsDNA, nucleosomes, citrullinated histone H4 and myeloperoxidase complexes were found to be significantly elevated and positively correlated with thrombin generation. In a transient middle cerebral artery occlusion model for stroke, administration of DNase I and neutralizing antibodies against histone led to smaller infracts^18^. Mechanistically, both neutrophils and platelets are known to activate each other either *via* P-selectin and β2/β3- integrins or cytokine/complement (IL-1β, TNF-α, GM-CSF, C3a, C5a) may mediate TREM-1 receptor interaction, which led to IL-8 release resulting in recruitment of neutrophils causing tissue injury^59^. Using human iliac artery biopsies, Wohner *et al*.^60^ demonstrated neutrophil derived elastase and metalloproteases degraded vWF and exposed promoting platelet adhesion^60^. In severe inflammatory conditions of sepsis, activated platelets induced NETs formation *via* TLR4^33^. On the other hand, histones and cfDNA released during NETs formation activated platelets leading to aggregation and coagulation suggesting interdependent activation of neutrophils and platelets leading to thrombosis^61^.

Thus we hypothesize that homocysteine induced NETs may result in interdependent activation of neutrophils and platelets leading to acceleration of thrombus formation during the pathogenesis of diabetes associated vascular diseases. Inability to fight infection especially in such cases as diabetes associated foot ulcers may also be due to dysfunctional neutrophils as a result of elevated homocysteine and IL-6 levels, and persistent hyperglycemic conditions. Homocysteine accelerated NETosis under hyperglycemic conditions may also suggest a connecting link between elevated serum homocysteine in T2D and its association with progression of vascular diseases.

## Materials and Methods

### Subjects and clinical characterization

We recruited age and gender matched 88 participants constituting healthy (n = 31, f/m = 16/15) and diabetic individuals (n = 57, f/m = 23/34) for this study which was approved by institutional ethical committee, Kasturba Hospital, Manipal University, Manipal. About 5 ml of peripheral blood was drawn with prior informed written consent for neutrophil and platelet isolation from healthy and diabetic individuals who visited outpatient Medicine department of Kasturba Hospital Manipal, India. Fasting and postprandial serum glucose levels of 126 mg/dl (7.0 mmol/l) and 200 mg/dl (11.1 mmol/l) respectively were defined as diabetic. Mean age of healthy control group was 49.58 ± 2.43 years and of diabetic individuals was 53.96 ± 1.4 years. Mean fasting glucose levels of control and diabetic subjects were 89.44 ± 10.40 mg/dL and 186.77 ± 74.21 mg/dL respectively. Mean post-prandial glucose levels of diabetic individuals were 241.83 ± 90.09 mg/dL. Diabetic control was assessed by glycated hemoglobin and mean HbA1c in diabetic group was 7.22 ± 3.96%. Body mass index of control (24.27 ± 3.3 kg/m^2^) and diabetic (26.66 ± 4.03 kg/m^2^) did not differ significantly. Mean total cholesterol, LDL, HDL and triglycerides of diabetic cohort were 156.38 ± 32.47 mg/dl, 91.23 ± 28.59 mg/dl, 36.14 ± 16.37 mg/dl and 143.95 ± 68.9 mg/dl respectively. 70% patients were treated with Biguanidine drugs (metformin), 20% patients were treated with sulfonylurea based therapy (Glibenclamide) and 10% patients were advised for dietary control. Diabetic patients who had prior history of systemic diseases (i.e., immunologic and auto-immune disorders, hypertension, mononuclear blood cell dysfunctions, infectious diseases, a history of cardio vascular disease, and other chronic systemic diseases) or treated with immunosuppressive medications were excluded from the study. All methods were conducted in accordance with the relevant guidelines and regulations.

### Neutrophil isolation

Neutrophils were isolated using well established Ficoll-Dextran method. Purified neutrophils were resuspended in neutrophil culture medium which comprised of RPMI 1640 medium supplemented with 2% heat inactivated human serum, 2 mM L-glutamine, 100 U/ml penicillin and 100 μg/ml streptomycin (Himedia, Mumbai, India). Neutrophil purity was assessed by Leishman’s staining and cell viability was 95–97% as determined by trypan blue dye exclusion method. CD11b staining using flow cytometry was performed to ensure that neutrophils were not activated during isolation.

### Neutrophil activation and quantification of NETs

NETs were quantified by staining extracellular DNA with membrane-impermeable DNA binding dye SYTOX Green (Molecular Probes, Invitrogen Life Technologies). Neutrophils (1 × 10^5^ cells) per well were seeded in 96 well plates and activated with homocysteine, phorbol −12-myrist −13 acetate, lipopolysaccharide (LPS). IL-6 (all from Sigma Aldrich, St Louis, MO, USA) along with untreated control and vehicle control for 3 hours. Diphenyleneiodonium (DPI), Ethylene glycol tetra acetic acid (EGTA), BAPTA-AM (Sigma Chemicals, USA) was used to assess role of NADPH oxidase and Ca^2+^ influx on NETosis respectively. After activation of neutrophils, SYTOX Green dye (5 μM) was added to each well and incubated for 15 minutes in dark. Fluorescence intensities were measured using Varioskan Flash (Thermo Scientific, Massachusetts, USA) at 480/530 nm.

### Immunocytochemistry

Neutrophils isolated from peripheral blood were seeded onto polystyrene coated chamber slides (ThermoFisher Scientific, MA, USA) and allowed to adhere. Cells were treated with homocysteine (100 μM) for three hours and washed once with PBS followed by fixing with 4% paraformaldehyde containing 0.1% Triton X 100 for 15 minutes. Further, cells were washed once with PBS and blocked with 5% BSA for one hour. Either anti-citrullinated histone or anti- myeloperoxidase antibody (both antibodies from Abcam, Cambridge, UK) were added at the concentration of 1:100 overnight at 4 °C. Slides were washed three times with 5% BSA and treated with TRITC labelled goat anti mouse antibody (Jackson Immunoresearch Laboratories, PA, USA). Cells were counterstained with DAPI (ThermoFisher Scientific, MA, USA). NETs were visualized in Olympus 1x-51 inverted fluorescence microscope with EMC2 Rolera camera and analyzed using image pro plus software v 7.0.

### Elastase assay

Neutrophils (10^5^cells/well) were treated with homocysteine for 3 hours and centrifuged at 600 × g for 10 minutes. The supernatant was subjected to DNase (8 U/ml) (Sigma Chemicals, USA) treatment for 20 minutes. Elastase was then measured using elastin substrate conjugated with BODIPY^@^ FL dye as per manufacturer’s instructions (EnzCheck Elastase assay kit; Life technologies, India.). Fluorescence was measured at 505/515 nm using Varioskan Flash (ThermoFisher Scientific, MA, USA).

### Measurement of mitochondrial superoxide

Mitochondrial superoxides in neutrophils were measured using MitoSOX dye (Molecular Probes, Life technology, Eugene, USA). Neutrophils (1 × 10^6)^ were preincubated MitoSOX for 30 minutes in dark at 37 °C, followed by inclusion of various activators of neutrophils as detailed in results and figure legends. Cells were washed once with PBS by centrifugation and accumulation of mitochondrial superoxide was measured in flow cytometry (BD FACS Caliber). Median intensity of superoxide radicals were quantified using Cell Quest Pro software.

### Neutrophil-platelet interaction

For co-culture assays, neutrophils were isolated as explained earlier. For platelet isolation, blood (3 ml) was collected and equal volume of citrate dextrose was added. The mixture was centrifuged at 190 g for 15 minutes at room temperature. The supernatant containing platelets were then centrifuged at 2500 g for 5 minutes at room temperature. The pellet was resuspended in RPMI media with 1% human serum and kept at room temperature for 2 minutes. Viability was checked with Trypan blue exclusion method. Platelets were activated with homocysteine in presence or absence of aspirin (Sigma chemicals, USA) for 30 minutes and washed with phosphate buffered saline and was subsequently co-cultured with neutrophils. The platelet-neutrophil ratio maintained at 1:10 and further incubated for 3 hours. NETs were evaluated upon staining with SYTOX Green using both fluorimeter and fluorescent microscope. Similar co-culture of neutrophils with untreated platelets served as negative control.

To assess influence of NETs on platelet aggregation, neutrophils (10^6^/ml cells) were seeded and stimulated with homocysteine for 30 minutes and washed gently by centrifugation. Neutrophils were resuspended in fresh medium in presence or absence of DNase (8 U/ml) and DPI (20 μM) for further 2.5 hours. Supernatant was collected by centrifugation and treated with platelets (10^7^/ml) in 96 well plate. Platelet aggregation was measured at 405 nm in Varioskan Flash plate reader (Thermo Scientific, Massachusetts, USA).

### ELISA

Platelets were isolated as described above and seeded at the density of 10^11^/ml in 96 well plates and treated with homocysteine for 15, 30 and 60 minutes. Protein levels of IL-6, IL-8 and TNF-α in platelet conditioned medium was measured by ELISA (Biolegend, CA, US).

### Immunoblotting

Neutrophils (10^6^/ml) were treated with or without homocysteine for different time points and lysed using 1% SDS with protease and phosphatase inhibitors followed by sonication at 40 Hz for 30 seconds. Cell lysates were mixed with Laemmli buffer and boiled at 90 °C for 10 minutes, resolved on a 10% SDS-PAGE and subsequently transferred to nitrocellulose membrane. Proteins were immunoblotted with corresponding primary antibody and detected with horseradish peroxidase-conjugated secondary antibodies and ECL (Pierce, ThermoFisher Scientific, MA, USA). Images of immunoblots were analyzed by ImageJ (NIH, USA).

### Quantification of plasma homocysteine

DL-Homocysteine levels in plasma of healthy and diabetic individuals were estimated by reversed-phase high performance liquid chromatography (RP-HPLC). In brief, 50 μL plasma along with 25 μL of internal standard (cysteinyl hydrochloride) and 25 μL phosphate buffered saline (PBS, pH 7.4) were incubated with 10 μL of 10% of tris (2-carboxyethyl) phosphine (TCEP) for 30 minutes at ambient temperature. For the purpose of deproteinization, 90 μL of 10% trichloroacetic acid (TCA) with 1 mmol/L EDTA was added. The mixture was thoroughly mixed and centrifuged at 13,000 g for 15 minutes and 50 μL of the supernatant was collected. A 10 μL of 1.55 mol/L NaOH; 125 μL of 0.125 mol/L borate buffers containing 4 mmol/L EDTA, pH 9.5; and 50 μL of 1 g/L SBD-F in the borate buffer were added to the collected supernatant. The samples were incubated for 60 minutes at 60 °C for derivatization and subjected to RP-HPLC (Waters 2695) on C18 column (Waters symmetry 250 x 4.6 mm, 5 μm) using 40 μL of sample injection volume with two buffers; 0.1 M potassium dihydrogen phosphate of pH 2.0 (Buffer A) and 0.1 M potassium dihydrogen phosphate of pH 2.0/ acetonitrile in the ratio 1:1 (Buffer B). The gradient of 88% buffer A and 12% of buffer B was optimized for mobile phase at a flow rate of 1.0 mL/minute using fluorescence detector set at excitation wavelength of 385 nm and emission wave length of 515 nm (Waters 2475). The generated HPLC peaks were analyzed using Empower II software.

### Estimation of cell free DNA in plasma

The levels of cell free (circulating) DNA was estimated from the plasma (25 μl) of both diabetic and controls using SYTOX Green dye (500 nM). Plasma and SYTOX Green dye were mixed and incubated in dark for 15 minutes at 37 °C and fluorescence was measured at 480/530 nm in Varisoskan Flash (Thermo Scientific, Massachusetts,USA). Standard graph was plotted using standard DNA (1 pg −1 μg) (Sigma chemicals, USA).

### Estimation of neutrophil elastase in plasma

Plasma levels of neutrophil elastase were estimated in diabetic patients and controls using Human PMN ELISA kit (Abcam, Cambridge, UK). Briefly, 50 μl of plasma was diluted following manufactures instructions and dispensed in precoated microtiter plate for one hour and followed by detection using coloring agent. Elastase levels were quantified in Varioskan Flash (Thermo Scientific, MA, USA) and absolute quantity was calculated using standard graph.

### Live imaging

Peripheral neutrophils (10^5 ^cells/well) from normal and diabetic individuals were stimulated with homocysteine (100 μM) along with SYTOX Green (5 μM). Images were captured every minute for 2 hours in Leica TCS SP8 confocal microscope (Leica Microsystems, Manheim, Germany) under 100 X original magnification. Images were processed using Leica Applications Suite X. For representing images fields with active NETosis were digitally zoomed. For fluorescence microscopy imaging of Ca^2+^, neutrophils were stained with Fluo3-AM (Thermo Fischer, MA, USA) and co-stained with DNA binding dye DAPI (Sigma Aldrich, St Louis, MO, USA) Images were captured in Olympus IX-51 inverted fluorescence microscope using EM-C2 Rolera camera (Q imaging, Surrey, Canada) and photomicrographs were analyzed by Image-Pro Plus software V 7.0.

### Statistical Analysis

All the experiments were performed in triplicate and repeated three times at different occasions and data are represented as mean ± standard error mean. Either two tailed student’s t-test or one way AMOVA with post-hoc Bonferroni multiple test was performed to calculate significant differences between experimental groups using Graphpad Prism 5. A P values less than 0.05 was considered as statistically significant. Linear correlation analysis using Pearson’s coefficient and stepwise multiple regression analysis was performed by R program (Stats package ver 3.0).

## Additional Information

**How to cite this article**: Joshi, M. B. *et al*. Elevated homocysteine levels in type 2 diabetes induce constitutive neutrophil extracellular traps. *Sci. Rep*. **6**, 36362; doi: 10.1038/srep36362 (2016).

**Publisher’s note:** Springer Nature remains neutral with regard to jurisdictional claims in published maps and institutional affiliations.

## Supplementary Material

Supplementary Information

## Figures and Tables

**Table 1:** Stepwise multiple regression analysis.

	Diabetes		All Subjects		Controls	
	R^2^ Value	p-Value	R^2^ Value	p-Value	R^2^ Value	p-Value
Fasting Glucose, Homocysteine, IL6	0.7031	3.47E-09	0.3812	4.19E-07	0.2194	0.0213
Homocysteine, IL6	0.6885	1.66E-09	0.3890	8.69E-08	0.2394	0.0083
Homocysteine	0.4789	1.77E-06	0.3272	3.87E-07	−0.0290	0.6976
IL6	0.5462	1.59E-07	0.2830	3.06E-06	0.2607	0.0020
