# Supplementary material for: Elevated homocysteine levels in type 2 diabetes induce constitutive neutrophil extracellular traps
Source: Sci Rep. 2016 Nov 4;6:36362. doi: 10.1038/srep36362 (PMC5095649; doi:10.1038/srep36362)

Elevated homocysteine levels in type 2 diabetes induce constitutive neutrophil extracellular traps

Manjunath B Joshi, Guruprasad Baipadithaya, Aswath Balakrishnan, Mangala Hegde, Manik Vohra, Rayees Ahamed, Shivashankara K Nagri, Lingadakai Ramachandra, Kapaettu Satyamoorthy

Figure S1. Correlation analysis between HbA1c and homocysteine, elastase, IL-6 and cfDNA.

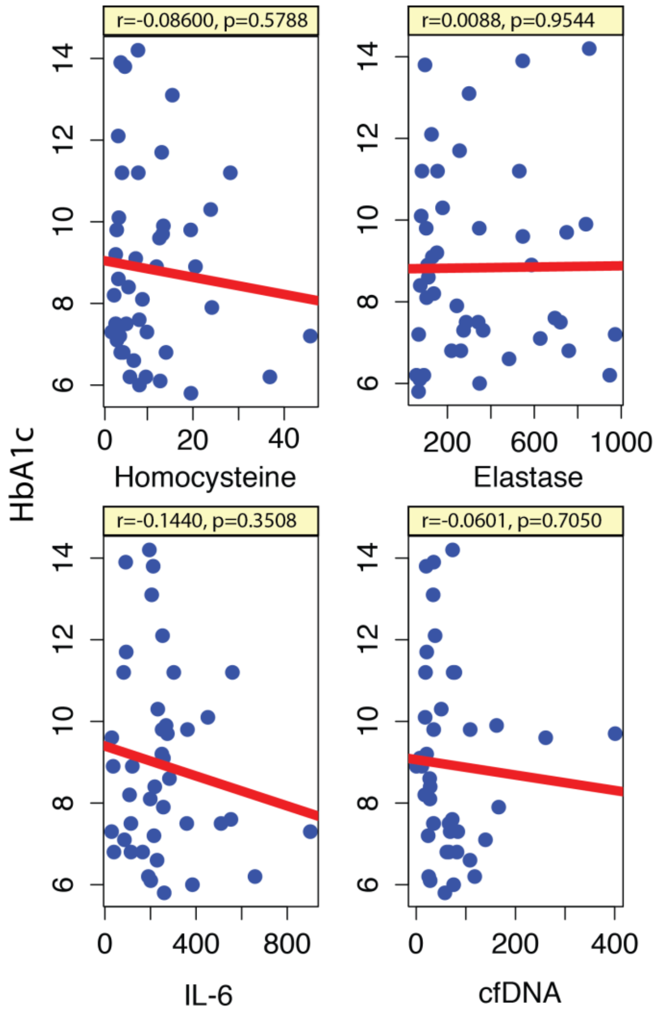

Supplement: Supplementary Information [file srep36362-s1.pdf]
